# Activation of the AT1R/HIF-1***α***/ACE Axis Mediates Angiotensin II-Induced VEGF Synthesis in Mesenchymal Stem Cells

**DOI:** 10.1155/2014/627380

**Published:** 2014-10-20

**Authors:** Chao Liu, Jing-Wen Zhang, Liang Hu, Yi-Chen Song, Lu Zhou, Yue Fan, Hong-Yi Zhu, Yu Wang, Qing-Ping Li

**Affiliations:** Collaborative Innovation Center for Cardiovascular Disease Translational Medicine, Department of Pharmacology, Nanjing Medical University, Nanjing 210029, China

## Abstract

A local renin-angiotensin system (RAS) is expressed in mesenchymal stem cells (MSCs) and regulates stem cell function. The local RAS influences the survival and tissue repairing ability of transplanted stem cells. We have previously reported that angiotensin II (Ang II) pretreatment can significantly increase vascular endothelial growth factor (VEGF) synthesis in MSCs through the ERK1/2 and Akt pathways via the Ang II receptor type 1 (AT1R). However, the role of angiotensin-converting enzyme (ACE) has not been clarified. Furthermore, whether Ang II pretreatment activates hypoxia-inducible factor-1*α* (HIF-1*α*) in MSCs has not been elucidated. Our data show that both ACE and HIF-1*α* are involved in promoting VEGF expression in MSCs, and that both are upregulated by Ang II stimulation. The upregulation of ACE appeared after the rapid degradation of exogenous Ang II, and led to the formation of endogenous Ang II. On the other hand, the ACE inhibitor, captopril, attenuated Ang II-enhanced HIF-1*α* upregulation, while HIF-1*α* suppression markedly attenuated ACE expression. This interesting finding suggests an interaction between ACE and HIF-1*α*. We conclude that Ang II pretreatment, as a trigger, activated the AT1R/HIF-1*α*/ACE axis that then mediated Ang II-induced VEGF synthesis in MSCs.

## 1. Introduction

A local renin-angiotensin system (RAS) has been reported to be expressed in rat mesenchymal stem cells (MSCs). The RAS components, angiotensin II receptors and angiotensin-converting enzyme (ACE), and the de novo synthesis of angiotensin II (Ang II) were detected in MSCs, suggesting that the local RAS could regulate MSC function by an autocrine-paracrine mechanism [1]. MSCs have recently been reported to be suitable for cell-based therapy of ischemic heart disease [2, 3]. The major mechanism of this therapy is a paracrine action [4, 5]. Our previous study revealed that Ang II pretreatment increases vascular endothelial growth factor (VEGF) synthesis in MSCs through the ERK1/2 and Akt pathways via the Ang II receptor type 1 (AT1R) [6]. However, the roles of other RAS components and their interactions in MSCs have not been clarified.

ACE is responsible for converting angiotensin I into Ang II. Numerous studies have demonstrated that ACE is involved in angiogenesis and VEGF expression in different tumor lines [7–9]. An antiangiogenic effect of ACE inhibitors has been observed in various cancer models, where ACE inhibitors have been shown to attenuate tumor growth and VEGF levels [10–12]. The role of ACE in Ang II-induced VEGF synthesis in MSCs is not known.

Hypoxia-inducible factor-1*α* (HIF-1*α*) regulates the expression of VEGF and other proangiogenic genes in response to hypoxia [13]. It is unstable under normoxic conditions and is stabilized under hypoxia [13]. Recent evidence suggests that some nonhypoxic stimuli, such as hormones, growth factors, and cytokines, activate HIF-1*α* in a cell-specific manner [14–18]. Therefore, we hypothesized that Ang II may stimulate VEGF expression via HIF-1*α* signaling in MSCs. HIF-1*α* is the upstream regulator of ACE in hypoxic human pulmonary artery smooth muscle cells (hPASMC) [19]. Whether HIF-1*α* influences ACE or other RAS components in MSCs has not been elucidated.

In the present study, we have investigated the roles of ACE and HIF-1*α* after Ang II pretreatment and revealed the signaling pathway by which ACE and HIF-1*α* mediate VEGF secretion in MSCs.

## 2. Materials and Methods

### 2.1. Ethics Statement

This study was approved by the Committee on the Ethics of Animal Experiments of Nanjing Medical University and complied with the recommendations in the Guide for the Care and Use of Laboratory Animals of the National Institutes of Health. All efforts were made to minimize the number of animals used and their suffering.

### 2.2. Reagents

Ang II and captopril were purchased from Sigma (St. Louis, MO, USA). Rat VEGF enzyme-linked immunospecific assay (ELISA) kit was from R&D systems (Minneapolis, MN, USA). Small interfering RNA was obtained from GenePharma (Shanghai, China). The Ang II radioimmunoassay kit (D02PJB) was provided by Beijing North Institute of Biological Technology (Beijing, China). The ACE activity kit was provided by Nanjing Jiancheng Bioengineering Institute (Nanjing, China). Antibodies raised against HIF-1*α* (Novus Biologicals, USA), ACE (Santa Cruz Biotechnology, USA), were used. Lipofectamine 2000 was purchased from Life Technologies (California, USA). Faststart Universal SYBR Green Master (ROX) was from Roche (Mannheim, Germany).

### 2.3. Isolation and Culture of MSCs

Male Sprague-Dawley (SD) rats weighing 60–80 g were provided by the Experimental Animal Center of Nanjing Medical University (Nanjing, China). Rats were killed by cervical dislocation. MSCs were generated by flushing the femurs with sterile DMEM (GIBCO, USA) and plated in 75 cm^2^ primary culture flasks with DMEM containing 10% fetal bovine serum (FBS, Hyclone, USA) as previously described [20]. Nonadherent cells were removed after 48 h and the media were replaced every 2 d for adherent cells. Each primary culture was passaged to new flasks when MSCs grew to approximately 80% confluence. Cells at passages two to five were used for the experiments.

### 2.4. Real-Time Quantitative PCR (qPCR)

Total RNA was extracted using the TRIzol reagent (Invitrogen Life Technologies, Gaithersburg, MD) and stored at −80°C. The SYBR Green Master was used according to the manufacturer's instructions. The primer sequences (sense/antisense) were as follows: VEGF, 5′-GCGGGCTGCTGCAATG-3′/5′-TGCAACGCGAGTCTGTGTTT-3′; ACE, 5′-ACGGAAGCATCACCAAGGAG-3′/5′-TGGCACATTCGCAGGAACG-3′; *β*-actin, 5′-GCACCGCAAATGCTTCTA-3′/5′-GGTCTTTACGGATGTCAACG-3′. The specificity of the amplification product was determined by performing a melting curve analysis. Standard curves were generated for the expression of each gene by using serial dilutions of known quantities of the corresponding cDNA gene template. Relative quantification of the signals was performed by normalizing the signals of different genes with the *β*-actin signal.

### 2.5. Western Blot Analysis

Whole-cell extracts were prepared using lysis buffer (1% Triton X-100, 20 mM HEPES [pH 7.5], 5 mM MgCl_2_, 1 mM EDTA, 1 mM EGTA, 1 mM dithiothreitol, 1 mM phenylmethylsulphonyl fluoride, and 1 mg/mL each of leupeptin, aprotinin, and pepstatin). Protein samples were separated on a 10% SDS-PAGE gel and transferred to a nitrocellulose membrane. The membrane was blocked in 5% milk/TBST and incubated overnight at 4°C with the following primary antibodies: ACE, HIF-1*α*, and *β*-actin. Membranes were exposed to HRP-conjugated secondary antibodies for 2 h at room temperature and protein expression was detected using enhanced chemiluminescence (ECL) detection reagent (Pierce).

### 2.6. ELISA Analysis

The MSC supernatants from all experimental groups were collected, centrifuged to remove cell debris, and stored at −80°C for analysis. VEGF concentration was measured according to the instructions of the ELISA kit. Spectrophotometric evaluation of VEGF levels was made using a Synergy HT multidetection microplate reader (BioTek).

### 2.7. siRNA Transfection

MSCs were seeded in 6-well plates. After 1 day, the cells were transfected with siRNA (100 nmol/L) using Lipofectamine 2000 according to the manufacturer's instructions. A corresponding random siRNA sequence was used as a negative control (Control). The siRNA sequences (sense/antisense) used were as follows: HIF-1*α* siRNA: 5′-GAG CUC CCA UCU UGA UAA ATT-3′/5′-UUU AUC AAG AUG GGA GCU CTT-3′; Control: 5′-UUC UCC GAA CGU GUC ACG UTT-3′/5′-ACG UGA CAC GUU CGG AGA ATT-3′. The effect of siRNA transfection was detected by western blot. Forty-eight hours after transfection, the cells were incubated in Ang II (100 nM) and collected in preparation for experiments.

### 2.8. Radioimmunoassay

The MSCs were seeded in 6-well plates and treated with 100 nM Ang II. The supernatants of pretreated MSCs were collected at different time points within 33 h and stored at −80°C for analysis. Supernatants of untreated MSCs were also collected. Ang II concentration was measured according to the radioimmunoassay kit instructions.

### 2.9. ACE Activity Measurement

ACE activity was measured according to the kit instructions. In brief, ACE activity was determined with an artificial substrate N-[3-(2-furyl)acryloyl]-L-phenylalanylglycylglycine (FAPGG) in a reaction mixture containing 25 *μ*L sample of MSC culture media and 100 *μ*L 2.5 mM FAPGG. Measurements were performed in 96-well plates at 37°C. Changes in optical density (340 nm) were measured at 5-minute intervals with a plate reader (BioTek). ACE activity was calculated according to the observed decrease in optical density.

### 2.10. Statistical Analysis

All data were expressed as mean ± SEM. Student's *t*-test was used for two-group comparisons and one-way ANOVA followed by Bonferroni correction was used for multiple group comparisons. A value of *P* < 0.05 was considered to be statistically significant.

## 3. Results

### 3.1. ACE Was Upregulated by Ang II Stimulation and Involved in VEGF Expression

**Figure 1:**
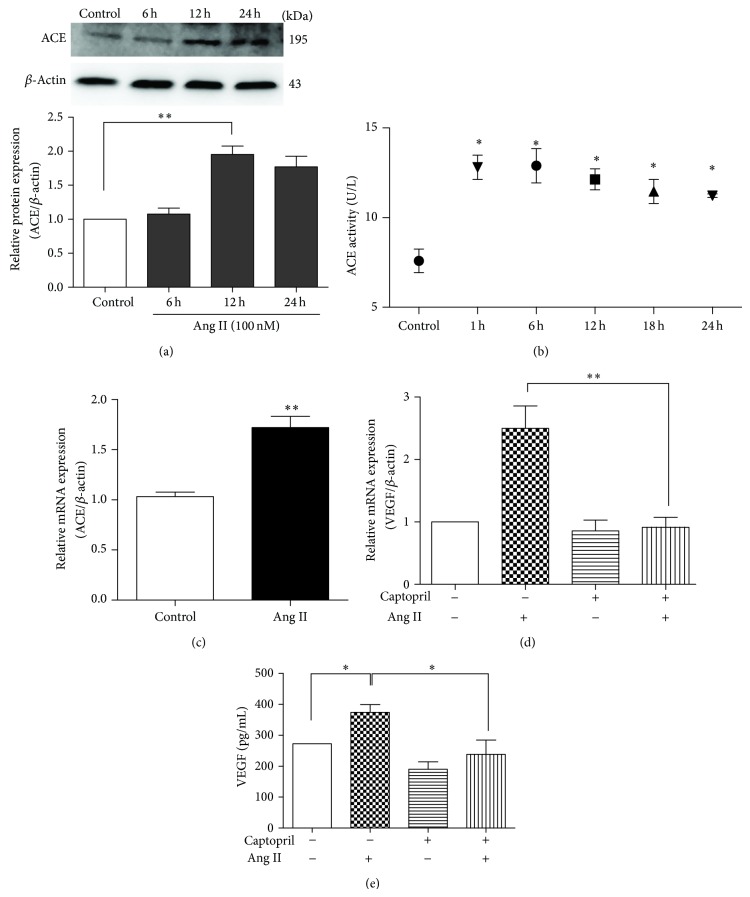
Upregulation of ACE after Ang II-induced VEGF synthesis. (a) Western blot analysis of ACE. MSCs were exposed to Ang II (100 nM) for 6 h, 12 h, or 24 h. *n* = 5, ^**^ *P* < 0.01. (b) Determination of ACE activity. MSCs were exposed to Ang II (100 nM) for 1 h, 6 h, 12 h, 18 h, or 24 h. *n* = 5, ^*^ *P* < 0.05 every other group* versus* control. (c) Real-time qPCR examination of ACE mRNA levels. MSCs were exposed to Ang II (100 nM) for 12 h. *n* = 5, ^**^ *P* < 0.01. (d) Real-time qPCR analysis of VEGF mRNA level, ^**^ *P* < 0.01. (e) ELISA of VEGF secretion. MSCs were pretreated with or without captopril (1 *μ*M for 1 h) before exposure to Ang II (100 nM) for 12 h. *n* = 5, ^*^ *P* < 0.05.

To determine the influence of Ang II stimulation on ACE, we examined ACE mRNA and protein expression. After exposure to 100 nM Ang II, ACE protein expression in MSCs doubled within 24 h in a time-dependent manner (*P* < 0.01; Figure 1(a)). Real-time qPCR showed that the ACE mRNA level in pretreated MSCs increased to over 1.5-fold of control (*P* < 0.01; Figure 1(c)). Additionally, Ang II stimulation induced 1.7-fold increase in ACE activity (*P* < 0.05; Figure 1(b)). To ascertain whether ACE was involved in Ang II-induced VEGF synthesis, MSCs were preincubated with 1 *μ*M captopril, a specific inhibitor of ACE, for 1 h before treatment with 100 nM Ang II for 12 h. VEGF mRNA expression and VEGF synthesis were then measured. After Ang II stimulation VEGF mRNA expression doubled. This effect was abolished by captopril (*P* < 0.01; Figure 1(d)). The Ang II-induced increase of VEGF secretion was also diminished in the captopril preincubation group (*P* < 0.05; Figure 1(e)). Therefore, we conclude that ACE promotes Ang II-induced VEGF expression in MSCs.

### 3.2. HIF-1*α* Was Upregulated by Ang II Stimulation and Involved in VEGF Expression

**Figure 2:**
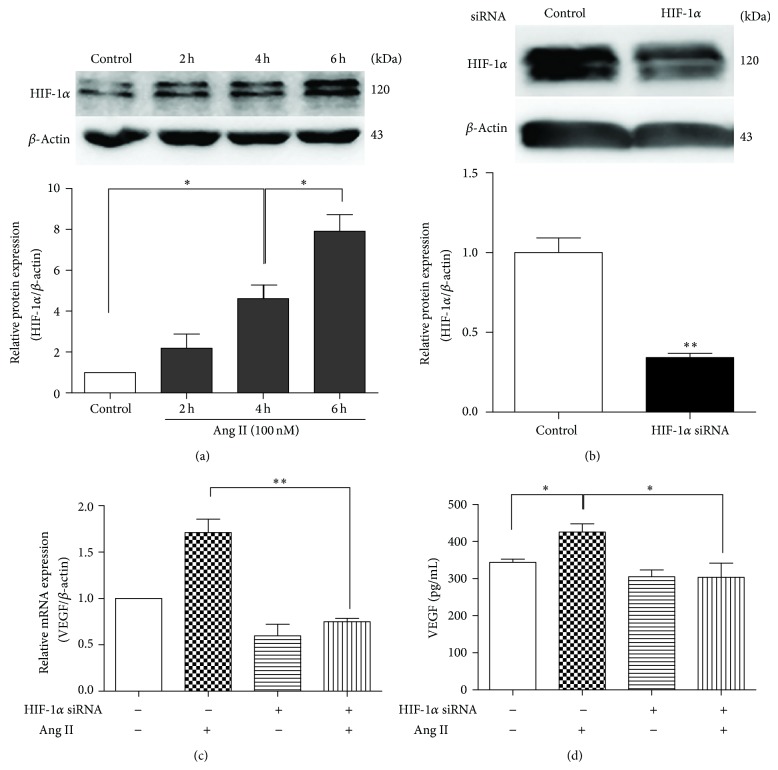
Activation of HIF-1*α* after Ang II-stimulated VEGF synthesis. (a) Western blot analysis of HIF-1*α* expression. MSCs were exposed to Ang II (100 nM) for 2 h, 4 h, or 6 h. *n* = 5, ^*^ *P* < 0.05. (b) Western blot analysis of HIF-1*α* siRNA efficiency. *n* = 3, ^**^ *P* < 0.01. (c) Real-time qPCR of VEGF mRNA level, ^**^ *P* < 0.01. (d) ELISA of VEGF secretion. MSCs were pretreated by HIF-1*α* siRNA before being exposed to Ang II (100 nM) for 12 h. *n* = 5. ^*^ *P* < 0.05.

To test the influence of Ang II stimulation on HIF-1*α*, we assessed HIF-1*α* protein expression after MSCs were exposed to 100 nM Ang II for 2, 4, or 6 h. HIF-1*α* was upregulated in a time-dependent manner (*P* < 0.05, Figure 2(a)). To determine whether the increase of VEGF was mediated by HIF-1*α*, MSCs were transfected with HIF-1*α* siRNA. The transfection group expressed less HIF-1*α* than the control group (Figure 2(b)). The Ang II-induced VEGF mRNA expression and protein secretion were markedly attenuated by knockdown of HIF-1*α* (*P* < 0.01, Figure 2(c); *P* < 0.05, Figure 2(d)). These results indicate that Ang II promotes VEGF expression via HIF-1*α* activation.

### 3.3. Interactions of HIF-1*α* and ACE Were Triggered by Exogenous Ang II

**Figure 3:**
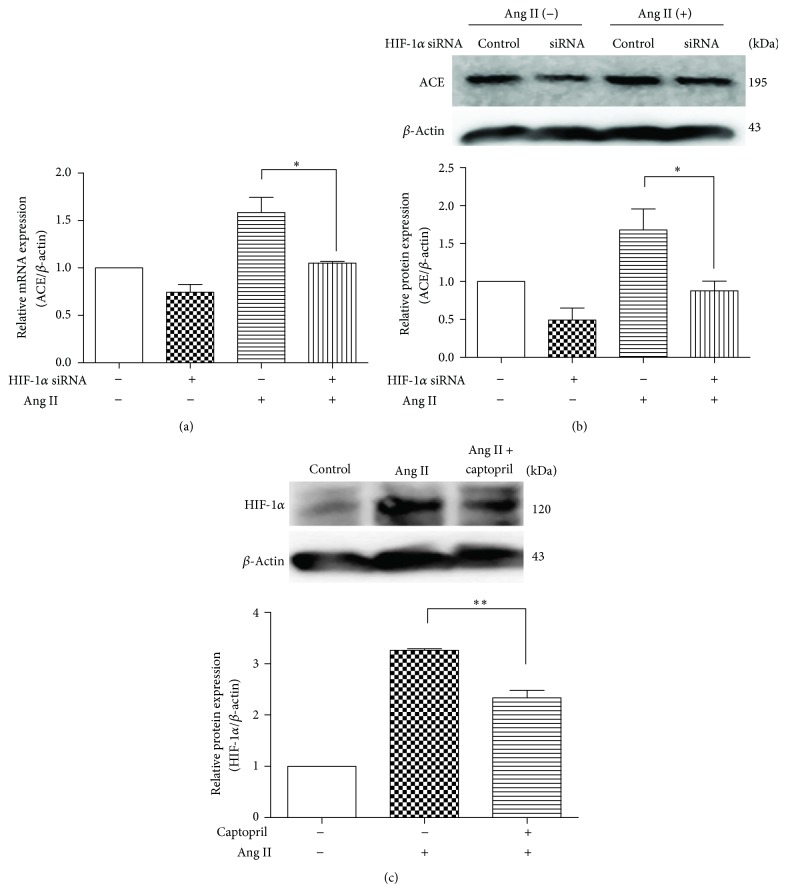
Interaction of HIF-1*α* with ACE in MSCs. (a) Real-time qPCR analysis of ACE mRNA level. MSCs were pretreated by HIF-1*α* siRNA before being exposed to Ang II (100 nM) for 12 h. *n* = 5, ^*^ *P* < 0.05. (b) Western blot analysis of ACE expression. MSCs were pretreated by HIF-1*α* siRNA before being exposed to Ang II (100 nM) for 6 h. *n* = 5, ^*^ *P* < 0.05. (c) Western blot analysis of HIF-1*α*. MSCs were pretreated with or without captopril (1 *μ*M) for 1 h before they were exposed to Ang II (100 nM) for 12 h. *n* = 5, ^**^ *P* < 0.01.

To study the potential interaction of HIF-1*α* with ACE, we inhibited HIF-1*α* by siRNA and ACE with captopril. Protein expression was analyzed by western blot. Transfection of MSCs with HIF-1*α* siRNA resulted in a significant decrease of ACE mRNA and protein expression (*P* < 0.05, Figure 3(a); *P* < 0.05, Figure 3(b)). Interestingly, a downregulation of HIF-1*α* protein expression was also observed when MSCs were pretreated with the ACE inhibitor, captopril (*P* < 0.01, Figure 3(c)). These results suggest that ACE and HIF-1*α* might interact with each other during their upregulation after MSCs are stimulated by Ang II.

### 3.4. Endogenous Ang II Was Augmented following ACE Upregulation after Pretreatment

**Figure 4:**
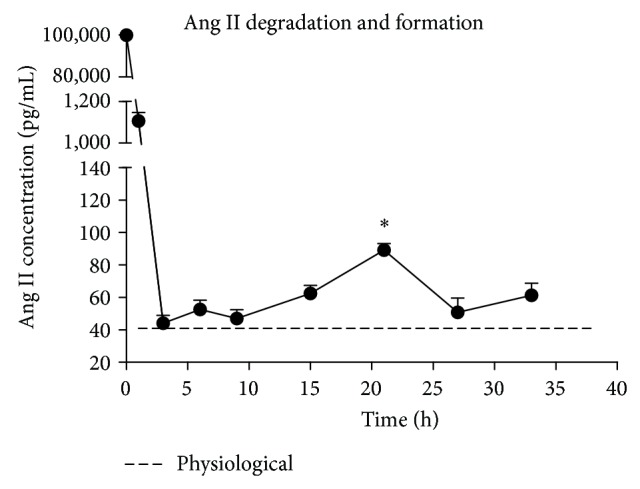
Ang II degradation and formation. Ang II concentration was determined by radioimmunoassay. Exogenous Ang II (100 nM) was added to the culture medium at time 0. Concentrations were determined at 1, 3, 6, 9, 15, 21, 27, and 33 h. *n* = 3. ^*^ *P* < 0.05, 21 h versus 3, 6, 9, 15 h. The concentration of Ang II in untreated MSC culture medium was also tested as the physiological concentration. *n* = 3.

The cleavage of exogenously added Ang II and the formation of endogenous Ang II in MSC culture media were determined by radioimmunoassay. Exogenous Ang II (100 nM) was added. The Ang II concentration was reduced to 1106 ± 40.48 pM after 1 h and continued to drop to 42.45 ± 4.26 pM after 3 h (Figure 4). However, the Ang II concentration increased significantly from 4 h (42.45 ± 4.26 pM) to 21 h (89.12 ± 4.02 pM) and then decreased at 27 h (50.78 ± 8.90 pM). The Ang II concentration tended to increase again from 28 to 33 h. The Ang II level of untreated MSCs, that is, the physiological concentration, was 41.05 ± 2.83 pM (Figure 4).

## 4. Discussion

The main findings of this study are that ACE and HIF-1*α* mediate Ang II-induced VEGF synthesis in MSCs and that ACE and HIF-1*α* appear to interact in this process. Both ACE and HIF-1*α* mediate the increase of VEGF synthesis by forming a positive feedback of the AT1R/HIF-1*α*/ACE axis.

**Figure 5:**
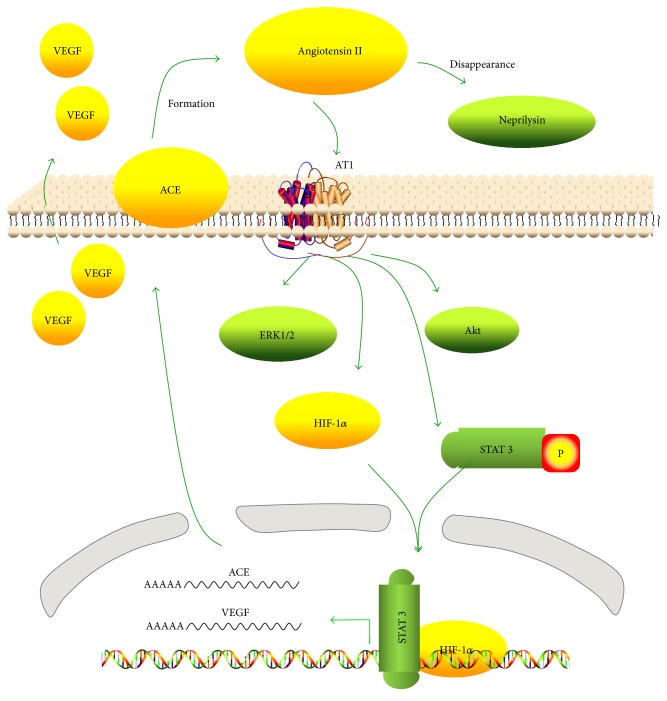
Scheme of Ang II-driven VEGF synthesis. Ang II induction sensing via HIF-1*α* or STAT3 activates an autocrine loop of ACE upregulation, Ang II formation, and signaling via AT1R, which exerts a positive feedback and further fosters VEGF synthesis in MSCs.

We have reported previously that Ang II stimulation could increase VEGF synthesis in MSCs through the ERK1/2 and Akt pathways via the AT1R [6]. This study examined the pathway that regulates ACE following activation of the AT1R by Ang II. Here, we report for the first time that ACE is upregulated after Ang II stimulation, and ACE upregulation also increases VEGF synthesis in MSCs (Figure 1). The upregulation of ACE appeared after the rapid degradation of exogenous Ang II and led to the formation of endogenous Ang II (Figure 4). Endogenous Ang II would be expected to continue to exert biological effects. We suggest that exogenous Ang II, as a trigger, induces VEGF synthesis with the formation of an Ang II-AT1R-ACE-VEGF autocrine system (Figure 5). In this process, exogenous Ang II is rapidly degraded. Neprilysin, an endopeptidase expressed on the cell surface, is the main enzyme responsible for degrading Ang II in preadipocytes and adipocytes [21]. Given the high activity of neprilysin in MSCs [22, 23], we attribute the rapid degradation of exogenous Ang II to neprilysin. A local RAS in MSCs was discovered 10 years ago [1]. The RAS components, angiotensinogen, renin, ACE, and AT receptors, were found to be present in cultured MSCs. The de novo synthesis of Ang II by MSCs was also detected. These findings demonstrated that a potential autocrine-paracrine mechanism existed in the local RAS of MSCs [1]. In this study, we also revealed that ACE activity of MSCs was raised by Ang II stimulation (Figure 1). Ang II concentration in culture media of MSCs increased from 4 h to 21 h (Figure 4), which was a result of the balance between ACE and neprilysin activities. In summary, a transient high level of exogenous Ang II acts as a trigger to upregulate ACE and induces a positive feedback of the RAS in MSCs. Although Schunkert et al. [24] reported Ang II infusion decreased ACE mRNA levels in the lung and testis of rats, later studies have confirmed a positive feedback effect on the local RAS with an ACE/AT1R-dependent mechanism in the kidney [25, 26].

HIF-1*α* is known to regulate VEGF expression in hypoxic conditions or after chemical stimulation. Several studies have indicated that HIF-1*α* can also be activated by Ang II [17, 27, 28]. In hPASMC, the phosphatidylinositol 3-kinase (PI3 K)/Akt pathway may mediate Ang II-stimulated HIF-1*α* upregulation [29]. However, the influence of Ang II pretreatment on HIF-1*α* signaling in MSCs has not been previously reported. In this study, we found for the first time that HIF-1*α* could be upregulated by Ang II stimulation and that the inhibition of HIF-1*α* significantly blocked VEGF enhancement by Ang II stimulation in MSCs (Figure 2). These findings suggest that HIF-1*α* is involved in Ang II-induced VEGF synthesis in MSCs.

To investigate the interaction of HIF-1*α* with ACE, we targeted siRNA to HIF-1*α* to inhibit its function and found that the inhibition of HIF-1*α* significantly blocked ACE upregulation (Figure 3). This finding indicates that HIF-1*α* acts as a mediator to promote ACE expression in MSCs. Additionally, when ACE was inhibited by captopril, Ang II-enhanced HIF-1*α* expression was significantly attenuated (Figure 3). This indicates that there is an interaction between HIF-1*α* and ACE-Ang II-AT1R signaling in MSCs. As shown in Figure 5, the data suggest that exogenous Ang II upregulates HIF-1*α* via the AT1R. Subsequently, HIF-1*α* not only activates VEGF expression, but also upregulates ACE expression which then increases endogenous Ang II formation and enhances AT1R activation.

Additionally, we have previously reported that Ang II-induced STAT3 phosphorylation activates VEGF mRNA expression and protein synthesis in MSCs [30]. An ACE inhibitor attenuated Ang II-induced STAT3 activation, while STAT3 inhibition diminished the upregulation of ACE. Considering these results, it is reasonable to speculate that the role of STAT3 is similar to that of HIF-1*α* in the fact that it not only influences VEGF mRNA expression but also activates ACE-Ang II-AT1R signaling. We have not examined the interaction between STAT3 and HIF-1*α* in MSCs. STAT3 is required for HIF-1*α* activation in human renal carcinoma cells and in female MSCs [31–33]. It can be inferred that STAT3 influences the AT1R-HIF-1*α*-ACE axis either by activating HIF-1*α* or by directly upregulating ACE, leading to increased VEGF expression (Figure 5).

In conclusion, the present results show that ACE and HIF-1*α* activation are involved in Ang II-induced upregulation of VEGF in MSCs. ACE and HIF-1*α* are involved in positive feedback of an AT1R-HIF-1*α*-ACE-Ang II loop. HIF-1*α* is possibly activated by STAT3 phosphorylation that is reported to cooperatively foster VEGF synthesis. These data clarify not only the mechanism of the Ang II stimulatory effect but also the mechanisms underlying Ang II-induced VEGF synthesis in MSCs. Our research sheds light on the cellular signaling underlying Ang II pretreatment for MSC transplantation. Further research in this area is underway. Under hypoxic conditions to mimic the infracted border-zone of the myocardium, the influence and mechanisms of pathologically elevated Ang II on the survival and paracrine effects of transplanted MSCs are under investigation. Also, we have preliminary results showing that transplantation of Ang II-pretreated MSCs resulted in better cardiac function than untreated MSCs.
